# Advances in macromolecular crystallography at the Photon Factory: automation from crystallization to structural determination

**DOI:** 10.1107/S1600577525001407

**Published:** 2025-04-04

**Authors:** Naohiro Matsugaki, Toshiya Senda

**Affiliations:** a Structural Biology Research Center, Photon Factory, Institute of Materials Structure Science High Energy Accelerator Research Organization (KEK) 1-1 Oho Tsukuba Japan; University of Manchester, United Kingdom

**Keywords:** Photon Factory, macromolecular crystallography, automation

## Abstract

The Structural Biology Research Center (SBRC) and the Photon Factory at the High Energy Accelerator Research Organization (KEK) have played a key role in advancing macromolecular crystallography (MX) and have developed advanced experimental systems in the MX field. Key innovations include a long-wavelength MX beamline for native single-wavelength anomalous diffraction phasing (BL-1A), a crystal-shaping machine, an automated crystal-centering system and a fully automated diffraction data-acquisition system. In addition to the beamline technologies, the SBRC has developed a fully automated protein crystallization and monitoring system (PXS/PXS2). The crystallization plate prepared by PXS2 can be mounted directly onto an *in situ* data-acquisition system at BL-17A. These technologies have transformed experimental workflows, enabling high-throughput structure determination and supporting drug discovery. Furthermore, the SBRC can integrate advanced imaging techniques, including MX, cryogenic electron microscopy (cryo-EM) and small-angle X-ray scattering (SAXS), under one roof. This interdisciplinary approach facilitates hybrid structural analysis by combining techniques such as MX and SAXS or MX and cryo-EM.

## Abstract 2

The Structural Biology Research Center at the High Energy Accelerator Research Organization has developed advanced systems for macromolecular crystallography, including an automated crystallization screening system, a long-wavelength crystallography beamline and an automated data-collection system.

## 1. Introduction – history of macromolecular crystallography at KEK

The Photon Factory (PF) at the High Energy Accelerator Research Organization (KEK), Japan’s first large-scale synchrotron facility, began operation in 1982. Since then, the PF has undergone three major upgrades to increase X-ray brightness and to create short straight sections (Katoh *et al.*, 1998 ▸; Honda *et al.*, 2005 ▸, 2007 ▸), and currently operates at an energy of 2.5 GeV. Short-gap undulators were later installed in the straight sections, some of which were used as light sources for macromolecular crystallography (MX) beamlines. KEK also manages another synchrotron radiation storage ring, the Photon Factory Advanced Ring (PF-AR), which runs primarily at 6.5 GeV in single-bunch mode.

The PF was built as a second-generation synchrotron facility and was quickly selected for various research activities in structural biology. Just like at other synchrotron facilities worldwide, including Stanford, Hamburg and Daresbury, these research activities expanded rapidly (Helliwell, 1992 ▸). The history of early synchrotron radiation use for MX is well documented by Hendrickson (2000 ▸). In the 1980s, Professors Sakabe and Satow developed MX data-collection systems. Professor Sakabe developed a Weissenberg camera for MX, which can have a much larger crystal oscillation angle than the typical oscillation camera (Sakabe, 1991 ▸; Sakabe *et al.*, 1995 ▸). Additionally, the Weissenberg camera employed an imaging plate (IP) (Fujifilm, Japan) instead of conventional X-ray films. The IP permitted rapid data readout via a dedicated image reader, drastically shortening data-collection time compared with X-ray films (Sakabe, 1991 ▸; Sakabe *et al.*, 1997 ▸). Data-processing software for the Weissenberg camera was written by Dr Higashi (Sakabe *et al.*, 1995 ▸). This camera attracted numerous researchers to the PF and contributed to the publication of many papers on MX. Consequently, several biologically important crystal structures were determined using the Weissenberg camera, including interferon-β, cytochrome c oxidase, ribonuclease H, thyroid hormone receptor and isoleucyl-transfer RNA synthetase (Senda *et al.*, 1992 ▸; Iwata *et al.*, 1995 ▸; Tsukihara *et al.*, 1995 ▸, 1996 ▸; Katayanagi *et al.*, 1990 ▸; Wagner *et al.*, 1995 ▸; Nureki *et al.*, 1998 ▸). The Weissenberg geometry was excellent for collecting diffraction data at that time. Since it allowed for a larger rotation angle per frame than the typical oscillation method, the number of IP films required for a dataset was greatly reduced. As a result, the data-collection time was significantly shorter compared with those by the four-circle diffractometer and the typical oscillation method. However, some theoretical disadvantages of this method remained. When unit cells were very large, the rotation angle had to be small to prevent overlapping diffraction spots. Additionally, large rotation angles resulted in high background noise. Despite these drawbacks, the Weissenberg method was effective in most cases, enabling phase determination through anomalous diffraction of heavy atoms such as mercury and platinum. Researchers from around the world visited the PF to use the Weissenberg camera. Unfortunately, due to the need for manual exchange of IP films and the long readout (and erasure) time, the method was not well suited to developments in the following decade – namely, the increased photon flux (which resulted in shorter exposure times) and beamline automation. Automated readout detectors, such as CCDs, were considered more suitable for emerging methods, which required collecting a larger amount of diffraction data from a single crystal.

At BL-14A, Professor Satow led another important effort by collaborating with Professor Hendrickson to collect multi-wavelength anomalous diffraction (MAD) data. This collaboration contributed significantly to establishing the MAD method as a new phasing technique (Phillips & Hodgson, 1980 ▸; Guss *et al.*, 1988 ▸; Hendrickson, 1991 ▸, 1999 ▸). The first PF application of the MAD method successfully determined the crystal structure of streptavidin (Hendrickson *et al.*, 1989 ▸), followed by the structures of interleukin-1, RNase H and others (Graves *et al.*, 1990 ▸; Yang *et al.*, 1990 ▸).

In 2000, Professor Wakatsuki joined the PF and introduced new insertion-device MX beamlines (AR-NW12A, BL-5A and BL-17A in 2003–2006) equipped with state-of-the-art CCD detectors (Quantum detector series; ADSC Inc.), further speeding up data collection. The existing bending-magnet MX beamlines (BL-18B, BL-6A) were decommissioned. Although IPs have a larger dynamic range than CCD detectors, CCD detectors have faster readouts, making them more suitable for shorter exposure times due to the increased photon flux from insertion-device light sources. This enables faster and more efficient data collection. Furthermore, for high-accuracy data collection, the fast-readout capability has made it possible to collect diffraction data with a smaller rotation angle to reduce background noise (Pflugrath, 1999 ▸). The implementation of CCD detectors accelerated MX experiments by eliminating the need to exchange the IP during diffraction data collection; researchers no longer had to enter the experimental hutch repeatedly to set the IP. As a result, data-collection speed increased by approximately three to four times. Importantly, the use of CCD detectors in combination with a sample-exchange robot (Hiraki *et al.*, 2007 ▸, 2008 ▸, 2013 ▸) enabled remote data acquisition, through the *PF Remote Monitoring System* (*PReMo*) software (Yamada *et al.*, 2008 ▸, 2013 ▸). We had a close collaboration with the SSRL in introducing sample changers in early 2000: most of the sample changers at the MX beamlines at PF are a variation of SAM (Stanford Automated Mounter) (Cohen *et al.*, 2002 ▸; Russi *et al.*, 2016 ▸). Some pharmaceutical companies then began using the remote diffraction data-collection system. In 2009, Astellas Pharma Inc., one of the biggest pharmaceutical companies in Japan, funded a dedicated high-throughput diffraction data-collection beamline at PF-AR (AR-NE3A) for structure-based drug discovery. Furthermore, in 2010, the Structural Biology Research Center (SBRC) established BL-1A, a long-wavelength MX beamline that enables native single-wavelength anomalous diffraction (SAD) phasing.

These activities after 2003 were carried out at the SBRC in collaboration with the PF and the Mechanical Engineering Center of KEK. The SBRC was established by Professor Wakatsuki in 2003 within the Institute of Materials Structure Science (IMSS). The SBRC’s primary roles were to support synchrotron radiation studies of biological macromolecules, advance technical developments and foster in-house structural biology research. In January 2013, Professor Senda joined KEK and became the director of the SBRC. This article provides an overview of MX activities at the SBRC. The SBRC presently comprises ∼40 members, with half focused on beamline operation and development and the remainder engaged in structural biology research, including cryogenic electron microscopy (cryo-EM).

## 2. Status of the PF

### 2.1. Status of MX beamlines

The SBRC operates five MX beamlines at the PF and PF-AR: BL-1A, BL-5A, BL-17A, AR-NW12A and AR-NE3A (Table 1 ▸). Each beamline is equipped with an insertion device, a dedicated sample changer and a pixel array detector (PAD), and has unique targets. A PAD is a photon-counting detector suitable for detecting weak X-ray signals with low readout noise and extremely short readout times. This enables shutterless and fine-phi-slice data collection. The PAD drastically reduced the data-collection time, and data quality improved due to the elimination of shutter jitter and the low background noise in fine-phi-sliced diffraction images (Mueller *et al.*, 2012 ▸). Thus, PADs are well suited for experiments requiring highly accurate data, such as native SAD phasing. Additionally, PADs have made raster scanning for identifying crystal positions a practical approach, which is essential for un­attended experiments.

BL-1A is designed for native SAD phasing with long-wavelength X-rays (2.7–3.5 Å) (see Section 2.6 for details). Long-wavelength X-rays can enhance anomalous signals of some light atoms, aiding in the identification of biologically significant light atoms such as Ca, S, Cl, P and K. BL-5A supports high-throughput and high-resolution data collection, achieving a maximum resolution of 0.68 Å. At BL-17A, users have access to an *in situ* data-collection system, allowing direct mounting of crystallization plates prepared by the PXS2 system (see Section 2.5 for details) in the biochemistry laboratory onto the goniometer. This beamline is also suitable for molecular replacement (MR)-native SAD data collection. AR-NW12A will soon include an online UV–Vis measurement system; an offline single-crystal spectroscopy setup is already available. AR-NE3A, built by a pharmaceutical company, is optimized for high-throughput data collection for drug discovery. The beamline is partially used by the company, while the remaining time is available to academic users.

Small-beam beamlines (BL-1A, BL-17A) are useful when sample crystals are smaller than ∼20 µm or when crystals are large but heterogeneous in quality. High-throughput beamlines provide a high-flux large beam, which is advantageous for larger homogeneous crystals, enabling high-speed data collection with shorter exposure times.

Researchers using the MX beamlines at the PF can select a beamline according to their experimental requirements. In principle, crystal dimensions and the need for anomalous scatterers determine the most suitable beamlines. Beamline staff are available for consultation on beamline selection. Briefly, BL-1A is suitable for native SAD phasing and for observing anomalous diffraction signals, such as those from Ca^2+^ and K^+^. For MR-native SAD phasing, both BL-1A and BL-17A are appropriate. Other beamlines are more suitable for high-throughput data collection with relatively large crystals. Importantly, the beamlines can be operated consistently through the *Unified Graphical User Interface* (*UGUI*) and *PReMo* (Yamada *et al.*, 2008 ▸, 2013 ▸) software. In 2023, ∼150 groups used the MX beamlines at PF and PF-AR, with diffraction data from these beamlines contributing to ∼300 structures deposited annually in the Protein Data Bank (PDB).

### 2.2. *UGUI* and *PReMo*

**Figure 1:**
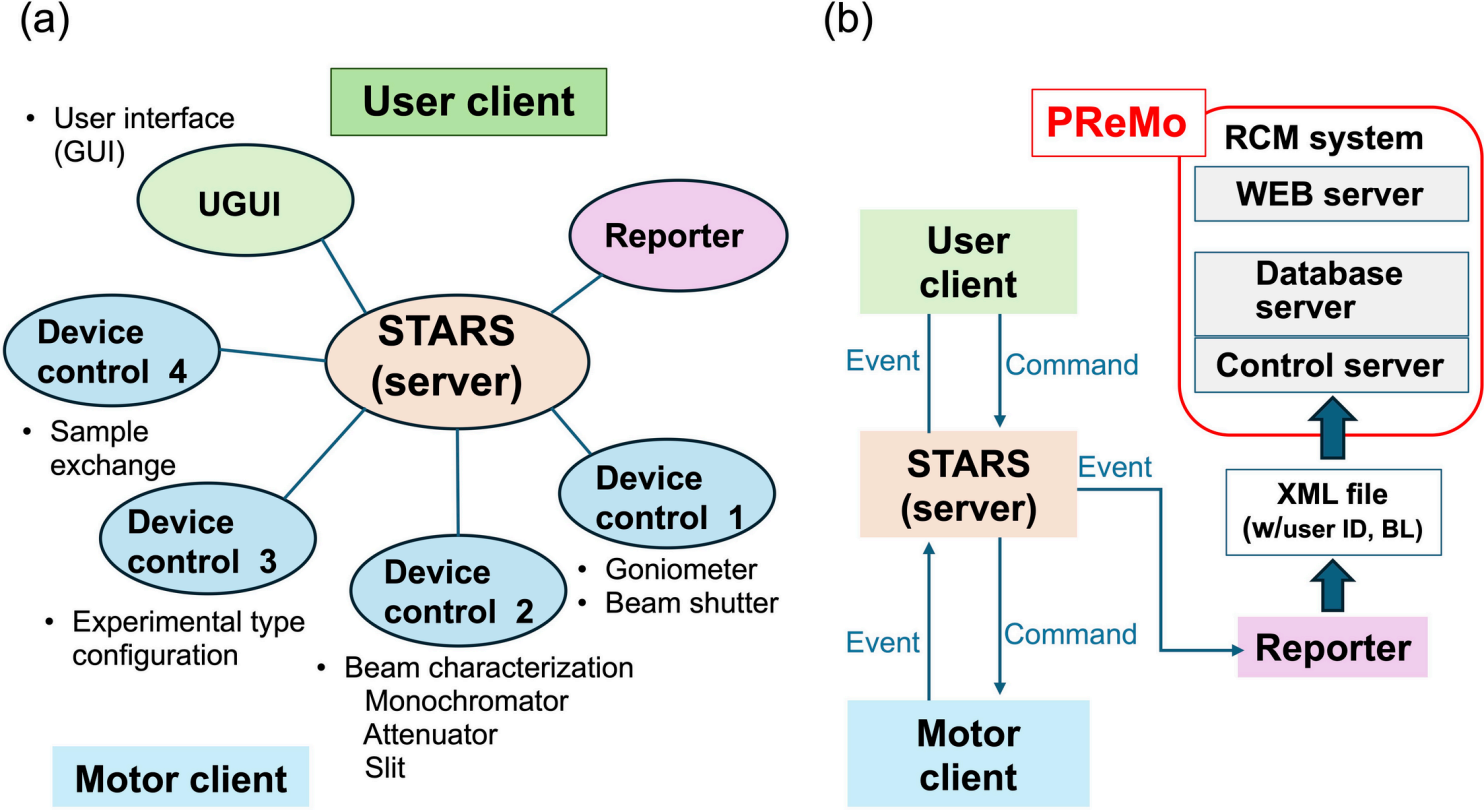
Beamline control system with STARS-based communication. (*a*) Schematic diagram of the client–server network for beamline control by STARS. (*b*) A diagram of the information flow. A ‘command’ is a text message transferred from the user client to the motor client(s) to direct individual experiments, and is followed by ‘event’ messages representing the latest status or positions of devices emitted from the motor client(s) to any clients related to the experiment. In this way the whole system is kept updated.

Each MX beamline is operated through *UGUI* software featuring GUIs for specific functions, such as adjusting X-ray energy, centering crystals, collecting diffraction images and configuring equipment for different experiment types. STARS (simple transmission and retrieval system) serves as the platform for text-based network communication (Kosuge *et al.*, 2002 ▸), enabling client software to exchange information via the STARS server. *UGUI* as a master client manages this process, enabling users to conduct individual experiments and execute predefined routine operations for unattended automated data collection [Fig. 1 ▸(*a*)]. *UGUI* provides a user interface for performing standard MAD/SAD or multiple-point data collection, with each point defined using multi-axis goniometry. Data-collection points can be booked in advance. More complex measurement sequences are possible using the built-in macro language, but this functionality is not available to general users.

*PReMo* (Yamada *et al.*, 2008 ▸, 2013 ▸) was initially developed as an experimental data management platform for the MX beamlines, using the commercial middleware *RCM* (R&D Chain Management) (QUATRE-i SCIENCE, Japan) as its foundation. Experimental data, in the form of XML files generated by each beamline’s reporting client software (*Reporter*), are stored in the *PReMo* database [Fig. 1 ▸(*b*)]. Through the web interface of *PReMo*, users can monitor the status of an experiment remotely. Additionally, *PReMo* enables automated data processing and analysis, such as phasing, model building and crystallographic refinement. Once a collection of diffraction data is completed, *PReMo* can trigger a data-processing job on a workstation cluster, followed by data analysis. All processing and analysis results are recorded in the *PReMo* database and are accessible via the web interface.

### 2.3. Automated data-collection system

Automation is crucial for efficient diffraction data collection. Many synchrotron facilities have developed their own automated data-collection systems (Wasserman *et al.*, 2012 ▸; Tsai *et al.*, 2013 ▸; Svensson *et al.*, 2015 ▸; Vollmar & Evans, 2021 ▸; Winter & McAuley, 2011 ▸; Hirata *et al.*, 2019 ▸; Smith *et al.*, 2023 ▸). At the PF, we have developed an automated data-collection system that includes a sample-exchange robot and automated crystal centering.

**Figure 2:**
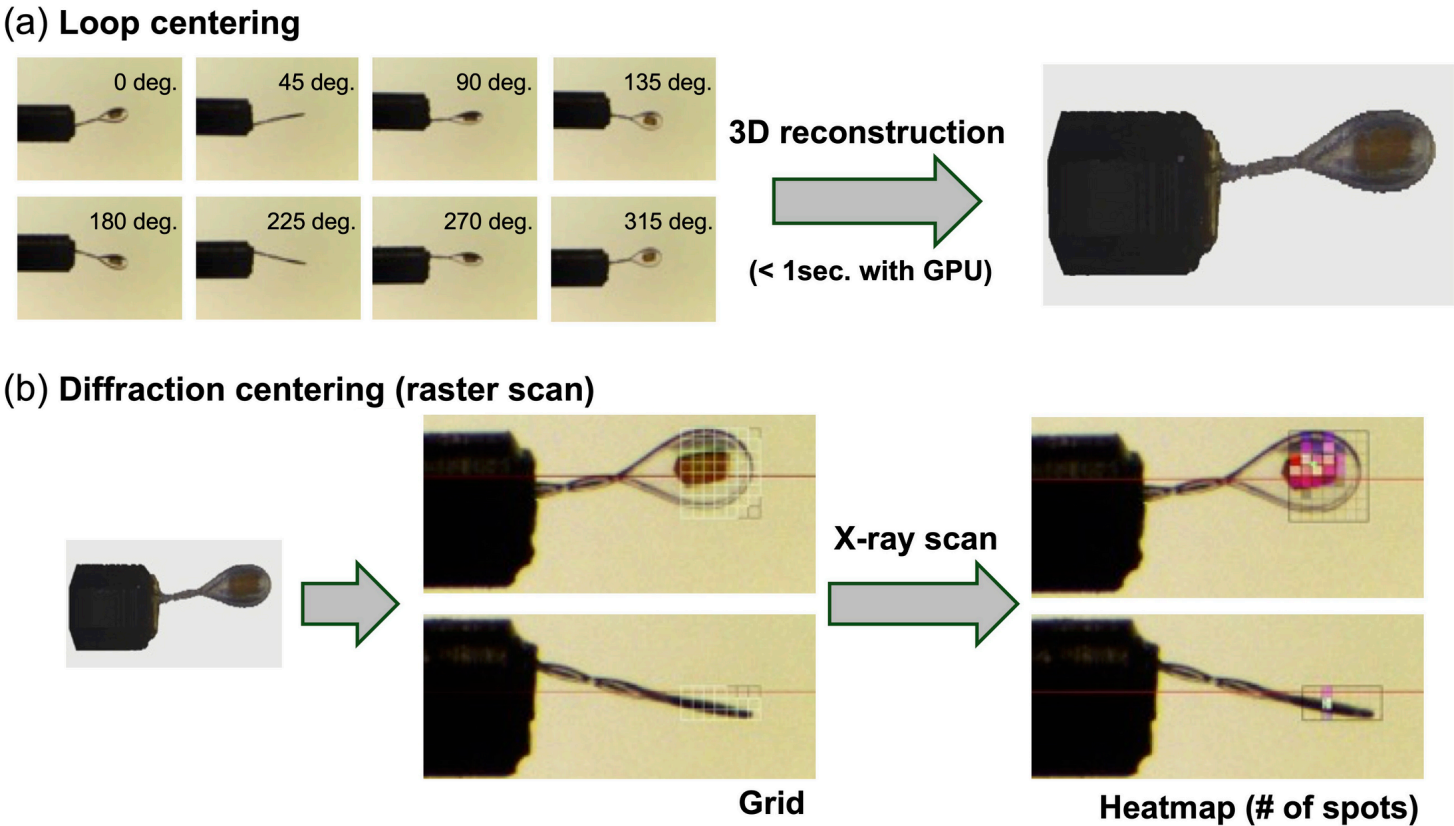
Two-step automated crystal centering. (*a*) Loop centering by 3D image reconstruction and (*b*) diffraction-based raster scanning to identify the sample crystal.

**Figure 3:**
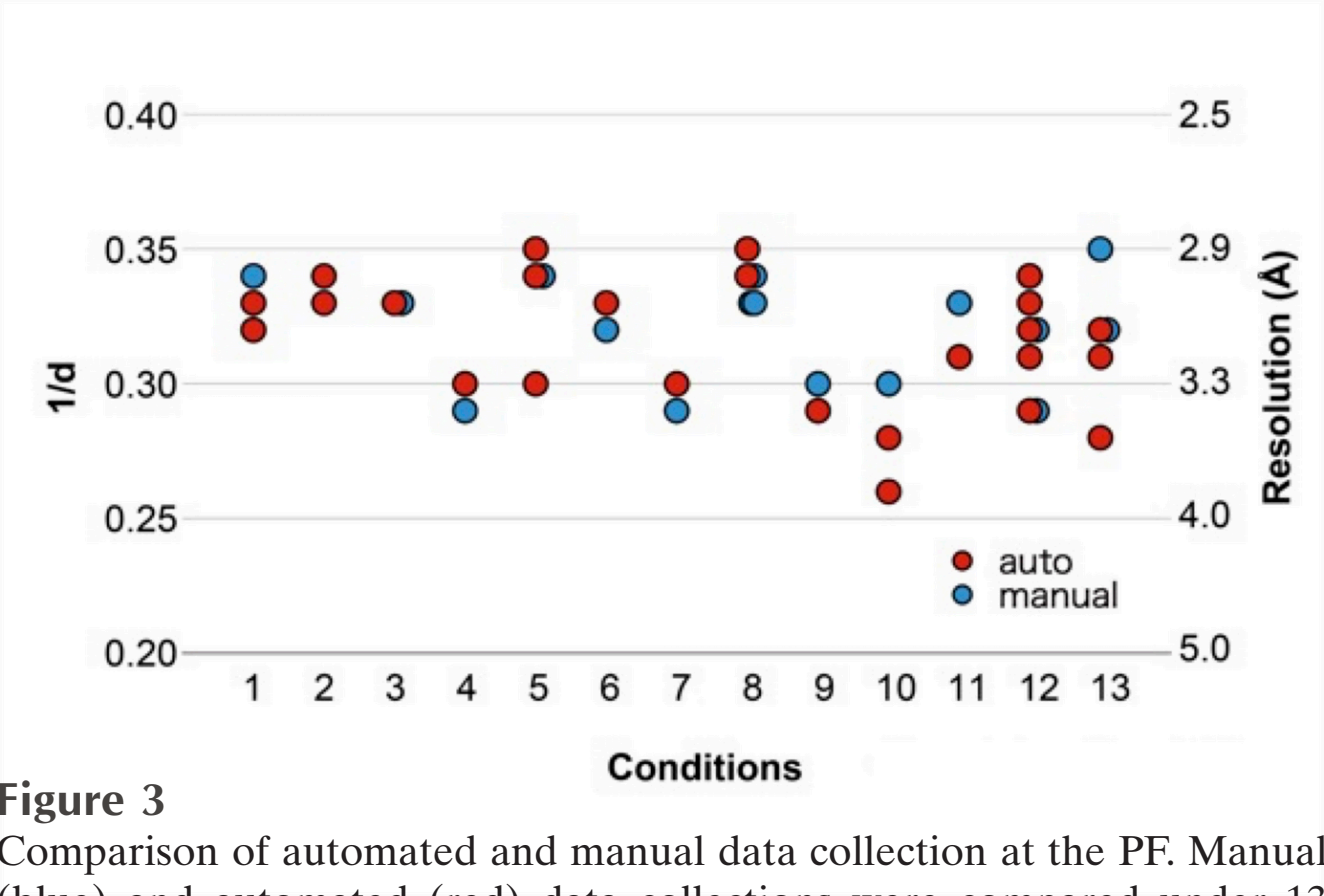
Comparison of automated and manual data collection at the PF. Manual (blue) and automated (red) data collections were compared under 13 different ligand-soaking conditions (nos. 1–13 on the horizontal axis) using crystals of the lipid kinase PI5P4Kβ. The crystals used in each condition were prepared under identical crystallization, cryoprotection and ligand-soaking conditions. Comparisons were based on the resolution at *I*/σ(*I*) values greater than 2.0. A single diffraction dataset was collected from each crystal, and no crystal was used more than once. No significant differences were observed between the automated and manual data collection, suggesting sufficient performance of the automated data collection.

All five beamlines in the PF are equipped with sample exchangers, and it has been possible to perform fully automated data collection with an automated loop-centering function since 2006. In 2017, we developed *SIROCC* (*Sophisticated Interface for Routine Operation with Crystal Centering*), a robust automated sample-centering system that identifies protein crystals in sample (or cryo-) loops through a two-step process (Fig. 2 ▸). First, *SIROCC* captures eight images from different angles, constructing a rough 3D model of the cryo-loop and crystal and estimating the cryo-loop’s orientation and region. Then, *SIROCC* performs X-ray raster scans with a fixed exposure time and low-dose X-ray in the estimated cryo-loop region. The number of diffraction spots below 4 Å resolution – typically considered to correspond to protein crystal diffraction – is used to determine the protein crystal’s position and shape. This method is effective even in the presence of ice around the mounted crystal. Although the obtained crystal size is not currently used for data-collection strategy, we plan to integrate it in the future. One limitation of this method is that raster-scan evaluation is based on the number of diffraction spots at each grid point. The number of diffraction spots may be overestimated when a crystal is cracked or when multiple crystals are stacked. *SIROCC*’s reliable sample centering was integrated into *UGUI* in 2017, enabling fully automated diffraction data collection beginning in 2018. So far, automated centering has been successful in more than 90% of cases, with most of the exceptions being cryo-loops with irregular shapes or sizes. Notably, our analysis showed no significant differences in data quality between fully automated and manual data collection (Fig. 3 ▸). Thus, the use of fully automated data collection is increasing.

To facilitate automated diffraction data collection, Rapid Access beam time is allocated at each beamline. Users with approved projects can reserve the Rapid Access beam time up to one week in advance of their desired usage schedule. Using this fully automated system, over 11000 datasets were collected in 2023. In addition, SIROCC was updated to enable multiple data collections from a heterogeneous crystal or multiple crystals within a single cryo-loop, further improving data-collection efficiency.

### 2.4. Automated crystallization screening with PXS/PXS2

While the automation system in the MX beamlines has streamlined diffraction data collection, crystallization remains the most challenging bottleneck in MX. To address this problem, the SBRC has developed an automated crystallization screening system parallel to beamline improvements.

The crystallization of biological macromolecules often involves extensive trial and error, requiring manual handling of solutions and microscopic inspection of each droplet. Although some commercial crystallization robots are available, manual intervention is frequently needed. To reduce the labor-intensive nature of crystallization screening and improve its efficiency and reliability, the SBRC developed a fully automated protein crystallization and monitoring system (PXS) in 2003 (Hiraki *et al.*, 2006 ▸). This system integrates solution dispensers, a plate-sealing robot, incubators and a droplet observation setup. Crystallization plates are transferred between components by software-controlled plate-carrying robots. After completing crystallization screening with multiple plates, the plates are placed in an incubator. PXS then automatically captures images of each droplet according to a programmed schedule. The captured droplet images are stored in an image server and can be reviewed using a dedicated GUI from anywhere in the world by accessing the server via the internet.

After more than a decade of PXS operation, user needs have changed; current users require crystallization screening with small amounts of protein samples rather than high-throughput screening. As a result, the PXS system was upgraded to PXS2 in 2014 by replacing most of the components (Kato *et al.*, 2021 ▸). We installed the Mosquito LCP (SPT Labtech, UK) for dispensing small samples. This machine can handle 0.1 µl of solution. In addition, the plate-carrying robot, plate sealer and observation system of the PXS were replaced with a robotic arm (Yasukawa, Japan), PS2002 plate sealer (Micronics, Japan) and RockImager 2 (Formulatrix, MA, USA), respectively. After the crystallization screening is completed, the crystallization plate is transferred to an incubator using another plate-carrying robot (Rorze, Japan). In PXS2, we have increased the number of automated incubators (20°C) (Rorze Lifescience, Japan), enabling us to store up to 1930 plates. We have also installed an incubator set at 4°C for low-temperature crystallization. However, when the plates stored at 4°C are observed with the RockImager 2 installed outside the incubator, the temperature difference leads to the formation of water droplets on the crystallization plates. Therefore, we installed another RockImager 2 inside the 4°C incubator. PXS2 can now handle volumes as small as 0.1 µl and can screen ∼800 conditions within 30 min in a single run, following a brief setup period. After completing one crystallization screening, the next screening can begin promptly when using the same crystallization solutions.

Recently, a Nikon microscope equipped with an electronic XY stage, electronic focus control and a high-resolution CCD camera was installed, enabling circularly polarized light observation for improved imaging and better recognition of small crystals of ∼1 µm in size. Along with the new imaging system, we have installed AI-based software developed in collaboration with AgroDesign Studios (Japan), capable of automatically scoring each droplet to determine whether it contains crystals. So far, less than 5% false positives and false negatives have been observed from 1440 wells of test crystallization plates, learned from more than 50000 training data points. Although crystallization droplets can be observed through a web application at our desks, manually checking over 800 droplets is labor intensive. Therefore, the automatic crystal-detection system greatly improves the crystallization screening experiments.

In addition to machine upgrades, two new functions were added to PXS2 (Kato *et al.*, 2021 ▸). The first is the ability to crystallize membrane proteins. PXS2 can perform bicelle and lipidic cubic phase (LCP) crystallization, meeting the demands of membrane protein studies in both academic and pharmaceutical research, with successful results already reported (Hayashi *et al.*, 2020 ▸). The second is an *in situ*X-ray diffraction setup at the synchrotron beamline (see below). Following crystallization screening on PXS2, users can mount a crystallization plate directly onto the BL-17A diffractometer to obtain diffraction images.

### 2.5. *In situ* data collection and PXS2 collaboration

**Figure 4:**
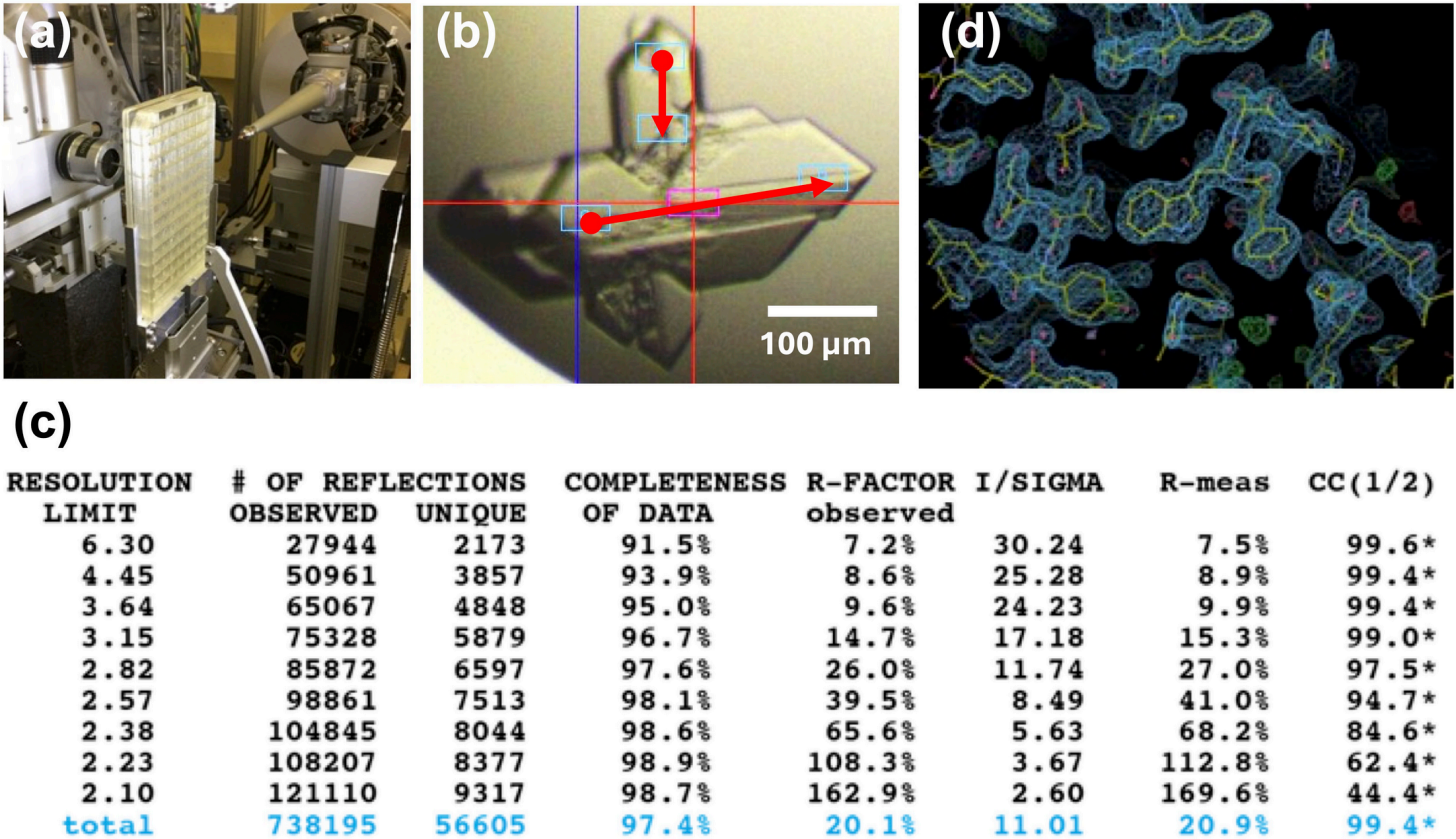
*In situ* data collection and structure solution. (*a*) The diffractometer at BL-17A for *in situ* data collection. (*b*) Helical data collection from multiple metalloprotein crystals to obtain complete datasets while reducing radiation damage. The plate was oscillated from −15° to +15° (30° in total), with an oscillation angle of 0.25° per frame and an exposure time of 0.05 s per frame. In total, 14 crystals from six drops were used. (*c*) Data-processing statistics of the helical data collection. The data were merged using *KAMO* (Yamashita *et al.*, 2018 ▸) with a maximum resolution of 2.1 Å (*I*/σ*I* > 2). (*d*) The 2*F*_o_ − *F*_c_ map of the protein solved using *in situ* data collection. The contour level is 3σ. The *R*_work _and *R*_free_ values are 0.18 and 0.23, respectively.

As mentioned above, we have developed an *in situ* system to collect diffraction data directly from a crystallization plate (Fig. 4 ▸). Conventional crystal handling – scooping individual crystals from a crystallization drop and mounting each of them onto a cryo-loop one at a time – is a time-consuming part of the MX experiment. Thus, this *in situ* system can significantly improve user experience. This system can also quickly assess both the content and quality of crystals (protein, salt, *etc.*) by using it in combination with PXS2. Another key application of this *in situ* system is that it allows data collection at room temperature.

To achieve *in situ* data collection, a dedicated goniometer was installed at BL-17A, allowing easy switching between a horizontal setting for conventional data collection and a vertical setting for *in situ* data collection [Fig. 4 ▸(*a*)]. Any type of crystallization plate in the Society of Biomolecular Screening (SBS) format can be mounted. However, a significant blind region exists due to the limitations of plate rotation. This limitation is addressed by measuring multiple crystals. Since crystals are randomly oriented in a crystallization droplet, a full dataset can be collected by acquiring diffraction data from multiple crystals. To reduce radiation damage at room temperature, helical data collection is available for *in situ* plate data collection. In addition, a plate-exchange robot enables data collection from multiple plates. Since the typical crystallization screening uses eight or nine plates, data collection from multiple plates is an essential feature for combined use with PXS2. A cooperative robot equipped with a dedicated gripper could retrieve a crystallization plate from a ‘plate hotel’ and mount it on the goniometer in the vertical setting. Remote-operation options are supported for plate mounting from the plate hotel.

After a crystallization plate is mounted, collection of diffraction data can start. For efficient use of the *in situ* data collection, one technical issue must be addressed. Since the rotation angles of the mounted plate are limited and the radiation damage during room-temperature data collection is high, randomly oriented tens of crystals may be required to gather a complete dataset. In the current system, we need to manually pick the crystal positions in the mounted plate before data collection, a rather time-consuming process. Figs. 4 ▸(*b*) and 4 ▸(*c*) show *in situ* multiple helical data collections of a metalloprotein. Each data collection was performed with a limited wedge of ∼30° to reduce radiation damage. Merging these partial datasets yielded a complete dataset that allowed structure determination by MR [Fig. 4 ▸(*d*)].

### 2.6. Long-wavelength MX beamline for native SAD phasing

Long-wavelength X-rays (generally longer than 2 Å) are advantageous in MX for applications such as native SAD phasing, light-atom identification, and model building at low resolution due to the enhanced anomalous signal from atoms such as sulfur and phospho­rus. Native SAD phasing is particularly attractive for *de novo* structure determination, as it avoids the need for derivative or seleno­methio­nylated crystals. However, due to weak anomalous signals, high-quality diffraction data are challenging to obtain. In addition, because these X-rays are easily absorbed by solvent, ice and the crystal itself, standard MX setups are inadequate to achieve the necessary data quality.

To address these challenges, we constructed BL-1A at PF in 2010, optimized for long-wavelength MX, specifically for native SAD phasing. The beamline was designed to deliver intense long-wavelength beams ranging from 2.7 to 3.5 Å with a beam size up to 13 µm (Table 1 ▸). The light source is a short-gap undulator located at one of the four short straight sections in the PF ring. The beamline is 20 m long from the light source to the sample position and consists of simple optics: a cryo-cooled channel-cut Si monochromator and a pair of bimorph KB focusing mirrors. To minimize long-wavelength beam loss, only one beryllium window separates the ring vacuum from the end station. The end station is equipped with a specially designed diffractometer for long-wavelength X-rays. Although the optimal wavelength for native SAD phasing was reported as ∼2.1 Å on standard MX beamlines (Mueller-Dieckmann *et al.*, 2005 ▸), severe X-ray absorption and air scattering along the beampath degrade the signal-to-noise ratio. To counter this, BL-1A features a helium chamber surrounding the diffractometer, complete with a helium-compatible goniometer, two X-ray detectors (two Eiger X4Ms), a cryostream of helium gas and a sample-exchange system. The BL-1A can therefore routinely support both long-wavelength experiments above 2.7 Å and standard low-background experiments near 1 Å without setup changes.

**Figure 5:**
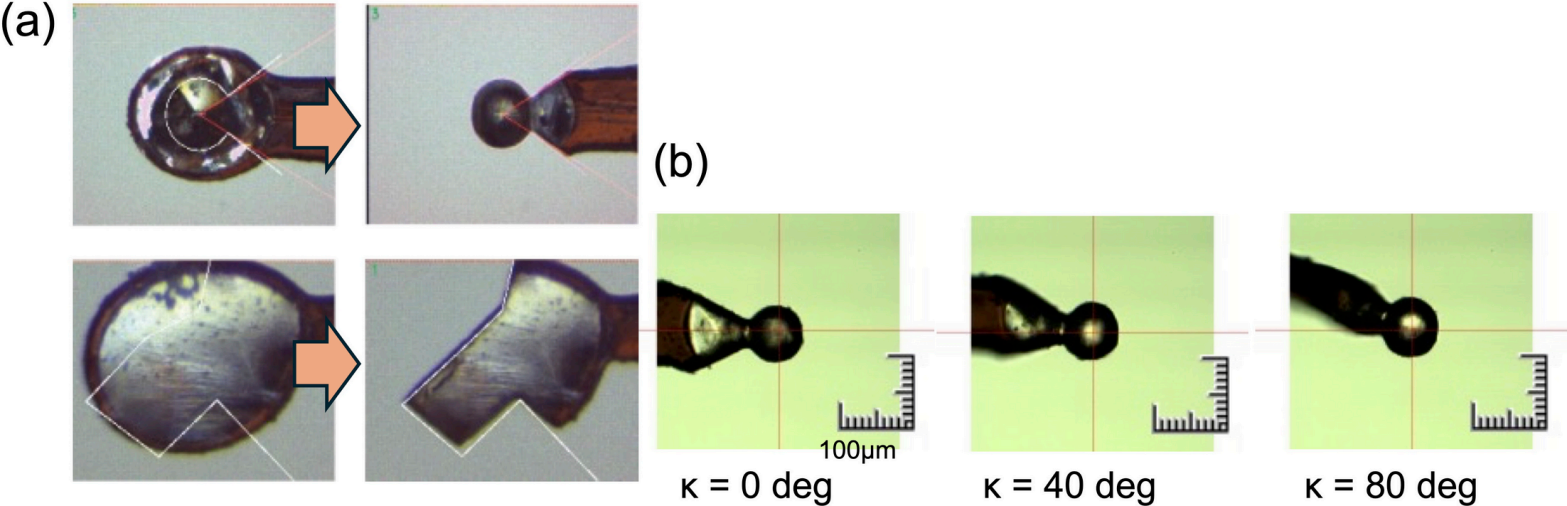
Crystal shaping with deep-UV laser. (*a*) Examples of crystal shaping – spherical (top) and rectangle shaping (bottom). (*b*) A spherical-shaped crystal of carbonic anhydrase from different kappa angle views used in native SAD data collection.

Due to sample absorption by the crystal and its surrounding medium, high-quality data collection remains a significant challenge, even when long-wavelength X-rays enhance the anomalous signals. The wavelength needed to maximize anomalous signals depends on crystal size (Liebschner *et al.*, 2016 ▸; Basu *et al.*, 2019 ▸), and the surrounding medium should be minimized to reduce background noise. To further optimize data collection, we introduced a crystal processing device using deep-UV laser ablation, originally developed at SPring-8 (Kawano *et al.*, 2022 ▸). This device allows precise removal of unwanted frozen sample areas and enables crystal shaping into spheres to reduce anisotropic absorption, which is difficult to correct computationally (Fig. 5 ▸). The integration of the crystal-shaping machine with BL-1A has resulted in many successful *de novo* structure determinations at the facility. Notably, carbonic anhydrase from *Anabaena sp.* PCC7120 (Hirakawa *et al.*, 2021 ▸), containing only one me­thio­nine residue in 174 residues (Bijvoet ratio = 0.86%), could be solved by merging 28 datasets from seven spherical crystals. The number of datasets for phasing could be reduced to eight (from two crystals) through the posterior analysis.

## 3. Future perspectives

### 3.1. Toward fully automated macromolecular crystallography

The SBRC has developed an automated diffraction data-collection system that has transformed the experimental workflow in MX. While automation has significantly advanced MX, further evolution is possible by integrating technologies developed at the SBRC. We have already automated systems for crystallization screening and *in situ* data collection, and these could be integrated with AI-based software that identify crystal positions within droplets. This integration would extend the fully automated MX system, encompassing both crystallization and data collection. While particular challenges remain, this goal is feasible.

Automated optimization of crystallization conditions is a critical next step. It can be achieved by leveraging accumulated experimental records and experience. Another necessary development is automated structure determination. Before *AlphaFold*, we considered that native SAD phasing could be a powerful method for automated structure determination. However, native SAD phasing is used less frequently as *AlphaFold* advances, since it has become possible to solve crystal structures using MR phasing with an *AlphaFold* model as a search model, even when no suitable coordinates for a search model are available in the PDB. However, because structure prediction is not perfect, model bias remains a concern. This issue can be mitigated by combining the MR method with the SAD method, which often yields a better electron-density map than MR alone. Since initial phases can be easily obtained using an *AlphaFold* model, combining MR and native SAD methods has become easier than before. Initial phases from MR methods are particularly useful for identifying sulfur atoms in the crystal, leading to significant improvements in SAD phasing with sulfur. Information on the sulfur positions is also helpful for molecular modeling during electron-density map interpretation, especially when the obtained electron-density map is of poor quality (Hayashi *et al.*, 2012 ▸). In addition, long-wavelength MX is useful for identifying biologically important light metal atoms, such as Ca^2+^ and K^+^. Since these atoms play crucial functional roles in proteins, determining their exact coordinates is often essential in biochemistry (Niwa *et al.*, 2014 ▸). However, applying this method to *in situ* data collection is challenging due to the relatively high background of the diffraction images caused by solvents and the crystallization plate. Although MR can be easily applied, MR-native SAD phasing provides an unbiased and high-quality electron-density map, facilitating automated model building. To improve data quality from *in situ* data collection, we are investigating alternative materials for PXS2 plates that can reduce background noise. Further studies will be essential to evaluate anomalous signal detection in *in situ* data collection.

### 3.2. Integrated structural biology

The SBRC is advancing integrated or hybrid structural biology methods, thereby enhancing our capabilities beyond MX. In addition to managing MX beamlines, the SBRC oversees two small-angle X-ray scattering (SAXS) beamlines, BL-15A2 and BL-10C, for bioSAXS studies. These beamlines are equipped with PADs and support the size-exclusion chromatography-SAXS (SEC-SAXS) method, enabling structural analysis of biological macromolecules in solution.

In parallel, the SBRC has initiated cryo-EM activities, like other synchrotron facilities such as eBIC at the Diamond Light Source (Saibil *et al.*, 2015 ▸). We installed our first cryo-EM instrument, a Talos Arctica (200 kV, Thermo Fisher Scientific), in 2017, followed by the construction of a dedicated cryo-EM laboratory in 2021 and the installation of a Titan Krios (300 kV) in 2022. With these resources, we are actively promoting the three-dimensional structural analysis of biological macromolecules by single-particle analysis with cryo-EM. Since many users are new to cryo-EM, the SBRC offers hands-on training covering both data acquisition and single-particle analysis. Today, nearly 50 research groups rely on KEK’s cryo-EM facilities for single-particle analysis of biological macromolecules. With the growing variety of 3D structure analysis methods, many users are now combining techniques, such as MX with bioSAXS or MX with cryo-EM, to enhance their structural insights (Adachi *et al.*, 2021 ▸; Katsuyama *et al.*, 2021 ▸; Mori *et al.*, 2021 ▸; Tao *et al.*, 2022 ▸). However, along with this expansion of cryo-EM activity, the lack of data-analysis expertise and computer resources has become a bottleneck for the method. We have been working to overcome these difficulties by developing a cloud-based computing environment using Amazon Web Service (AWS) (Moriya *et al.*, 2024 ▸). The problem of limited access to sample-preparation/grid-screening facilities will be mitigated by introducing an additional dedicated 100 kV microscope.

While hybrid 3D structural analysis has become more common, the SBRC’s integration of MX, bioSAXS and cryo-EM under one roof, along with its in-house expertise in each area, offers unique advantages for users pursuing hybrid analyses. Our interdisciplinary team provide comprehensive consultation, drawing on their expertise to assist users across multiple methods and deliver high-quality user support.

Looking forward, the SBRC plans to expand into imaging fields such as X-ray computed tomography (CT) using synchrotron radiation and cryo-electron tomography (cryo-ET), thus enabling trans-scale structural analysis. At the PF, X-ray imaging beamlines have also been developed. For example, BL-14 is a unique beamline equipped with a vertical wiggler that enables biological X-ray imaging techniques such as phase-contrast X-ray CT. Although the beamline has been mainly used to observe the morphology of biological samples, there is likely to be a growing demand for X-ray imaging in the field of life sciences. As gene-editing techniques have made it easier than ever to generate knock-in/knock-out animals, X-ray imaging has become increasingly important for understanding the relationship between morphology and atomic resolution biochemical processes in cells. To meet this need, the SBRC is planning to develop a bioimaging beamline for a wide range of life science researchers. In addition, cryo-ET has become a critical tool for observing cellular structures, including organelles. We consider the introduction of a cryo-ET facility a key objective for the SBRC in achieving integrated structural biology. As the life sciences shift from reductionist approaches to integrative studies, structural biology must also evolve to link molecular functions with larger biological contexts, from cells to tissues and organs. To support this vision, the SBRC is committed to advancing imaging technologies with synchrotron radiation, positioning trans-scale structural analysis as a next-generation tool for structural biology.

## Figures and Tables

**Table 1:** Specifications of the MX beamlines at the PF

	Small beam		High throughput		
Beamline	BL-1A	BL-17A	BL-5A	AR-NW12A	AR-NE3A
Starting year	2010	2006	2004	2003	2009
Wavelength (Å)	0.96–1.03, 1.2–1.9, 2.7–3.5	0.9–2.1	0.75–1.9	0.75–1.9	0.75–1.9
Typical beam size (H × V, µm)	13 × 13	40 × 16	200 × 200	200 × 130	Ø100
Photon flux (photons s^−1^)	2.5 × 10^10^ (@1.03 Å)	2.1 × 10^11^ (@0.98 Å)	3.0 × 10^11^ (@1.0 Å)	2.5 × 10^11^ (@1.0 Å)	4.2 × 10^11^ (@1.0 Å)
Detector	Eiger X4M (×2)	Eiger X16M	Pilatus3 S6M	Pilatus3 S2M	Pilatus 2M-F
Maximum resolution (Å)	3.0 (@ 2.7 Å)	0.88 (@ 0.98 Å)	0.68 (@ 0.75 Å)	0.78 (@ 0.75 Å)	0.76 (@ 0.75 Å)
Sample exchanger	PAM-HC (Unipuck)	PAM (Unipuck, SSRL)			
Goniometer	Mini-κ on a horizontal rotation axis	Single rotation axis (horizontal)			
Target	Native SAD	Native SAD *in situ*	High resolution	Spectroscopy	Fully automated
